# Cracking failure of curved hollow tree trunks

**DOI:** 10.1098/rsos.200203

**Published:** 2020-03-11

**Authors:** Yan-San Huang, Pei-Lin Chiang, Ying-Chuan Kao, Fu-Lan Hsu, Jia-Yang Juang

**Affiliations:** 1 Department of Forestry, National Chung Hsing University, 145 Xingda Road, South Dist., Taichung City 402, Taiwan; 2 Department of Mechanical Engineering, National Taiwan University, Taipei 10617, Taiwan; 3 Division of Forest Chemistry, Taiwan Forestry Research Institute, 53 Nanhai Road, Taipei 10066, Taiwan

**Keywords:** bending failure, cross-sectional flattening, tangential crack, curved hollow trunk, orthotropic material

## Abstract

Understanding the failure modes of curved hollow tree trunks is essential from both safety and conservation perspectives. Despite extensive research, the underlying mechanism that determines the cracking failure of curved hollow tree trunks remains unclear due to the lack of theoretical analysis that considers both the initial curvature and orthotropic material properties. Here we derive new mathematical expressions for predicting the bending moment, *M*_crack_, at which the cracking failure occurs. The failure mode of a tree species is then determined, as a function of *t*/*R* and *cR*, by comparing *M*_crack_ with *M*_bend_, where *t*, *R* and *c* are, respectively, the trunk wall thickness, outer radius and initial curvature; *M*_bend_ is the bending moment for conventional bending failure. Our equation shows that *M*_crack_ is proportional to the tangential tensile strength of wood *σ_T_*, increases with *t*/*R*, and decreases with the final *cR*. We analyse 11 tree species and find that hardwoods are more likely to fail in conventional bending, whereas softwoods tend to break due to cracking. This is due to the softwoods' much smaller tangential tensile strength, as observed from the data of 66 hardwoods and 43 softwoods. For larger *cR*, cracking failure is easier to occur in curvature-decreasing bending than curvature-increasing due to additional normal tensile force *F* acting on the neutral cross-section; on the other hand, for smaller *cR*, bending failure is easier to occur due to decreased final curvature. Our formulae are applicable to other natural and man-made curved hollow beams with orthotropic material properties. Our findings provide insights for those managing trees in urban situations and those managing for conservation of hollow-dependent fauna in both urban and rural settings.

## 1. Introduction

Slender hollow structures have the merit of resisting bending moment and torque with a relatively lower weight per unit length than solid cylinders of the same weight [1]. They are ubiquitous in nature and are commonly seen in many organisms as a result of convergent evolution. Examples include decayed hollow tree trunks [2–4], bamboo stems, cereal stalks [5], porcupine quills [6], animal bones [7] and microtubules [8]. Many living trees have their cores rotten and become hollow, known as piping [2]. For instance, 37% of trees, belonging to a wide range of species, were found to be hollow in a study conducted in the Amazonian rainforest [3]. In this paper, we focus on ‘tree trunks’; however, our methods generalize to any slender hollow structure with orthotropic material properties. Symbols are listed in table 1.

**Table 1.** Nomenclature.

notation	
*c*	initial curvature of a trunk
*cR*	dimensionless initial curvature of a trunk
$c_{cri}R$	critical *cR* value at *M*_crack_ = *M*_bend_
*c*_min_*R*	the minimum *cR* for which cracking can occur—cracking does not occur for *cR* < *c*_min_*R*. *c*_min_*R* = 2*K*. Only applicable for the case of decreasing curvature
$c_{}^{\prime }$	change of curvature due to bending moment $M_{c^{\prime }}$. $c^{\prime }=M_{c^{\prime }}/E_{L}I$
$c_{}^{\prime }R$	dimensionless change of curvature due to bending moment $M_{c^{\prime }}$
$c_{\mathrm{bend}}^{\prime }R$	critical $c_{}^{\prime }R$ at which conventional bending failure occurs, i.e. $M_{c^{\prime }}=M_{\mathrm{bend}}$. $c_{\mathrm{bend}}^{\prime }R=\sigma_{b}/E_{L}$
$c_{\mathrm{crack}}^{\prime }R$	critical $c_{}^{\prime }R$ at which cracking failure occurs, i.e. $M_{c^{\prime }}=M_{\mathrm{crack}}$. $c_{\mathrm{crack}}^{\prime }R\leq K$
*E_L_*	Young's modulus in the longitudinal direction
*I*	moment of inertia of the cross-section of a trunk
*K*	$K\equiv \sqrt{(c\pm {c^{\prime }}_{\mathrm{crack}}){c^{\prime }}_{\mathrm{crack}}}R$ at $M_{c^{\prime }}=M_{\mathrm{crack}}$. ‘+’ and ‘−’ are respectively for increasing and decreasing curvatures. $K=c_{\mathrm{crack}}^{\prime }R$ for straight trunks
*Q*	$Q\equiv c_{cri}R\pm c_{\mathrm{crack}}^{\prime }R$ at *M*_bend_ = *M*_crack_.‘+’ and ‘−’ are respectively for increasing and decreasing curvatures. *Q*/*R* is the final curvature at failure
*M*_bend_	bending moment at which the conventional bending failure occurs
*M*_Brazier_	bending moment at which the Brazier buckling failure occurs—a thin-walled tube, subjected to bending moment, buckles because the cross-section ovalizes
*M*_crack_	bending moment at which the tangential cracking occurs
*M*_crack0_	bending moment at which the tangential cracking occurs for a straight trunk
$M_{c^{\prime }}$	bending moment that creates a curvature change of $c_{}^{\prime }$
*R*	outer radius of a hollow trunk
*t*	wall thickness of a hollow trunk
(*t*/*R*)_*cri*_	critical *t*/*R* ratio at which *Q* = *K*. *c_cri_R*, or equivalently *M*_crack_ = *M*_bend_, exists if and only if *t*/*R* ≥ (*t*/*R*)*_cri_*
*ε_L-failure_*	normal strain at which the conventional bending failure occurs. *ε_L-failure_* = *σ_b_/E_L_*
*σ_b_*	bending strength in the longitudinal direction
*σ_T_*	tangential component of tensile strength perpendicular to fibres, or tangential tensile strength for short

**Figure 1.**
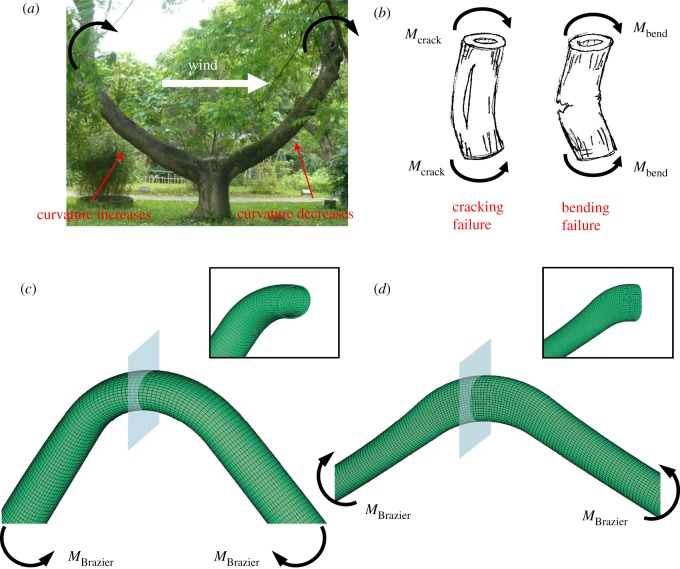
(*a*) An example of curved tree trunks: the outer radius *R* ≈ 0.2 m, the initial curvature *c* ≈ 0.35 m^−1^ and the dimensionless initial curvature *cR* ≈ 0.07; *Trema orientailis* (hardwood). Photo credit: Yan-San Huang. (*b*) Schematics of cracking and bending failures. (Trunk sketches by Da-Chang Yang.) (*c*,*d*) Finite-element simulations of cross-sectional flattening, known as Brazier buckling, of a curved circular hollow trunk subjected to curvature-increasing bending (*c*) and curvature-decreasing bending (*d*). The cross-section becomes ovalized with its long axis perpendicular to the plane of bending in case (*c*), or parallel to the plane of bending in case (*d*). *cR* = 0.2, *R* = 0.1 m, *c* = 2 m^−1^ and *t* = 0.005 m.

When a tree is subjected to strong wind, the wind-induced drag force acts on the crown and trunk, and causes a large bending moment on the trunk and root plate. For an initially curved tree trunk, the bending moment may increase or decrease its curvature, depending on the wind direction (figure 1*a*). This moment is the main source of trunk failure.

Solid- or thick-walled hollow tree trunks tend to break due to conventional bending failure—fibres buckling on the compression side, followed by fibres tearing on the tension side (figure 1*b*). The cross-section remains circular until the break, and the classic bending theory is suitable for predicting the maximum bending moment the trunk can resist. Thin-walled trunks, however, tend to break due to cracking failure—tangential crack initiation on the inner surface, followed by longitudinal splitting (figure 1*b*). Cracking failure is due to cross-sectional flattening or ovalization [5,9–13], a phenomenon neglected by the classic bending theory. The mechanism of cross-sectional flattening is briefly described as follows. When subjected to a bending moment, an elastic straight beam forms a curve. The fibres on the convex and the concave sides are, respectively, under tensile and compressive stresses in the axial direction. Those longitudinal tensile and compressive stresses also set up transverse compressive forces, pointing toward the neutral axis, on both convex and concave sides [12,14]. These forces then compress the circular cross-section to become oval with the long axis perpendicular to the plane of bending. For isotropic ductile materials, such as metals or plastic drinking straws, the cross-section buckles and eventually collapses without cracking, known as Brazier buckling failure [15,16]. For an initially curved trunk subjected to curvature-increasing bending, the orientation of the ovalized cross-section is the same as that of a straight trunk (figure 1*c*); however, in curvature-decreasing bending, the orientation is different and has the long axis parallel to the plane of bending (figure 1*d*).

The exact failure mode of a trunk depends on its material properties and geometric parameters. The former includes (i) the ratio *t/R* of wall thickness *t* to outer radius *R*, (ii) the initial curvature *c*; the latter includes Young's modulus *E*, bending strength in the longitudinal direction *σ_b_*, and tangential component of tensile strength perpendicular to fibres *σ_T_*, called tangential tensile strength hereafter. It also depends on the wind direction if the trunk is initially curved.

Wood is a natural orthotropic material, with fibres generally aligned along the tree axis, that has different mechanical properties in the three mutually perpendicular axes—longitudinal, radial and tangential. The longitudinal axis is parallel to the fibres; the radial and tangential axes are on the cross-section perpendicular to the fibres. Since the fibres are longitudinally oriented, Young's modulus and breaking strength are the highest in the longitudinal direction [17] and are approximately 15-fold to 20-fold larger than those in the tangential direction [18,19]. These orthotropic material properties must be taken into consideration when predicting the failure modes.

Spatz & Niklas [5] successfully used numerical simulations to predict the critical bending moments of various failure modes and considered *t*/*R* ratio, slenderness and orthotropic material properties. Numerical simulations, although powerful, may sometimes come at the detriment of physical insights and analytical understanding of the interplay between key parameters. Our earlier work presented an analytical expression for predicting the bending moment for tangential cracking of straight hollow trunks [13]. Using Taiwan red cypress as an example, we showed that Brazier buckling, cracking failure and conventional bending failure occur for 0 < *t*/*R* < 0.06, 0.06 < *t*/*R* < 0.27 and 0.27 < *t*/*R* < 1, respectively. Since trunks with extremely small *t*/*R* are rare, only the bending and cracking failures are relevant.

Despite the extensive studies, there is still no analytical expression for predicting the cracking failure for initially curved orthotropic trunks, and our understanding on how they fail is still lacking. In this paper, we follow the theoretical framework in [13] to derive such analytical expressions for both curvature-increasing and curvature-decreasing bending. We apply the expressions to analyse the failure modes of 11 tree species, including four hardwoods, three softwoods and four tropical woods.

Specific gravity is the most important physical property of wood which influences its strength as long as the wood is sound [20]. In general, hardwoods have larger specific gravity than softwoods. We also compare the material properties, as a function of specific gravity, between hardwoods and softwoods for 66 species of hardwoods and 43 species of softwoods.

## 2. Theoretical formulation

**Figure 2.**
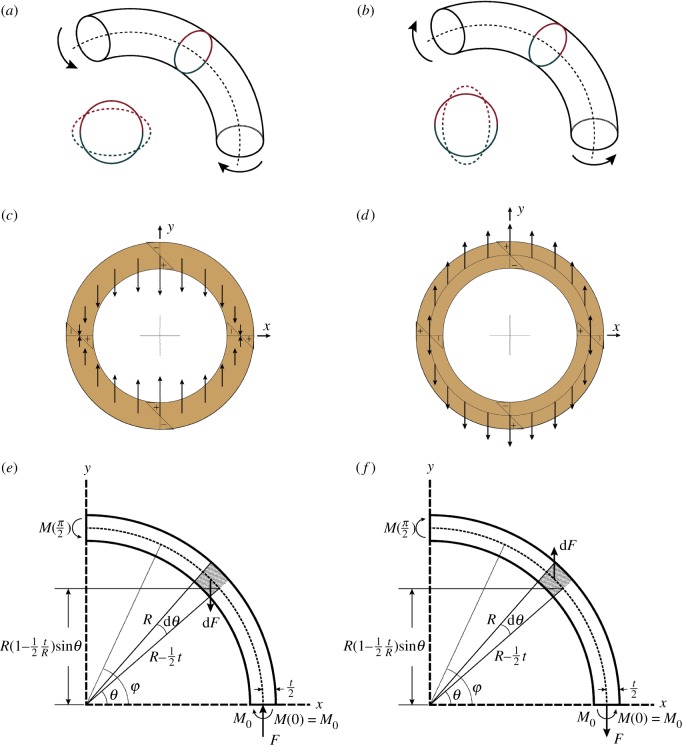
Analysis of cross-sectional flattening of a curved circular hollow trunk for curvature-increasing (*a*,*c*,*e*) and for curvature-decreasing (*b*,*d*,*f*). (*c*,*d*) Inward and outward force distribution in a transverse section. (*e*,*f*) Free-body diagram of one quarter of the transverse section for calculating the bending moment *M*(*φ*) exerted on axial sections with an angular position *φ*. *θ*, angular position of the transverse force d*F*; *M*_0_, statically indeterminate bending moment at *φ* = 0. The direction of d*F* determines the direction of cross-sectional flattening. Note that d*F* is a body force per unit axial length (unit: N m^−1^), not a shear force. *F* is the internal normal force per unit length acting on the axial-radial plane at *φ* = 0.

Figure 2 shows the schematics of curved hollow trunks subject to curvature-increasing bending (figure 2*a,c*,*e*) and curvature-decreasing bending (figure 2*b*,*d*,*f*), respectively. The circular cross-sections are flattened (figure 2*a*,*b*) due to the transverse distributed forces (figure 2*c*,*d*) set up by the longitudinal bending stresses. These transverse forces induce tangential moments acting on axial-radial planes (figure 2*e*,*f*), which creates tangential cracking if the tensile stress exceeds the tangential tensile strength *σ_T_*.

We determine the failure mode at a given *t*/*R* ratio and *cR* by comparing the magnitudes of bending moment at which the conventional bending failure (*M*_bend_) and tangential cracking failure (*M*_crack_) occur. The expression for *M*_bend_ is obtained from the literature, and those for *M*_crack_ for curved trunks derived here are new and have not been reported before. We derive the equation for the change of curvature, corresponding to the bending failure ($c_{\mathrm{bend}}^{\prime }$) and cracking failure ($c_{\mathrm{crack}}^{\prime }$), and also the equation for the critical *cR* = *c*_*cri*_*R*, as a function of *t*/*R*, at which *M*_bend_ = *M*_crack_.

According to the classical bending theory, the maximum bending moment of a tree trunk is

2.1 $$M_{\mathrm{bend}}=\frac{\sigma_{b}I}{R},$$

where *σ_b_* is the bending strength of typical green wood in the longitudinal direction, *I* is the cross-sectional moment of inertia and *R* is the outer radius of the circular cross-section. For a hollow trunk with small wall thickness to radius ratio, *t/R*, *M*_bend_ = *πR*^3^(*t*/*R*)*σ_b_*, and for a solid trunk (*t*/*R* = 1), the bending moment is $M_{\mathrm{bend}\_\mathrm{solid}}=\pi R^{3}\sigma_{b}/4$.

### 2.1. Initially curved hollow trunk subject to curvature-increasing bending

#### 2.1.1. Derivation of *m*_crack_ for cracking failure

For an initially curved trunk with a circular hollow cross-section, the tangential cracking is initiated at *φ* = *π*/2 when subjected to curvature-increasing bending. The corresponding bending moment *M*_crack_ is derived as follows (electronic supplementary material):

2.2 $$M_{\mathrm{crack}}=\frac{2\sigma_{T}I(t/R)}{3(cR+{c^{\prime }}_{\mathrm{crack}}R)R{\left(1-(1/2)(t/R)\right)}^{3}}\;\text{for }\varphi =\frac{\pi }{2},$$

where *cR* is the dimensionless initial curvature, and $c_{\mathrm{crack}}^{\prime }R$ is the dimensionless change of curvature when cracking failure occurs, which will be determined in the following section.

#### 2.1.2. Derivation of *K* and $c_{\mathrm{crack}}^{\prime }R$ for cracking failure

If an initially curved trunk is subject to a curvature-increasing bending moment $M_{c^{\prime }}$, its curvature will increase by $c_{}^{\prime }$

2.3 $$c^{\prime }=\frac{M_{c^{\prime }}}{E_{L}I},$$

where *E_L_* is Young's modulus in the longitudinal direction.

From equations (2.2) and (2.3),

2.4 $$\frac{M_{c^{\prime }}}{M_{\mathrm{crack}}}=\frac{3(cR+{c^{\prime }}_{\mathrm{crack}}R){c^{\prime }}_{}RE_{L}{\left(1-(1/2)\;(t/R)\right)}^{3}}{2\sigma_{T}(t/R)}\;\text{for }\varphi =\frac{\pi }{2}.$$

For crack failure initiation, $M_{c^{\prime }}=M_{\mathrm{crack}}$ and $c_{}^{\prime }R=c_{\mathrm{crack}}^{\prime }R$. Define $K\equiv \sqrt{(cR+{c^{\prime }}_{\mathrm{crack}}R){c^{\prime }}_{\mathrm{crack}}R}$ and from equation (2.4)

2.5 $$K=\frac{\sqrt{2\sigma_{T}(t/R)}}{\sqrt{3E_{L}{\left(1-(1/2)\;(t/R)\right)}^{3}}}.$$

Solving $K^{2}=(cR+c_{\mathrm{crack}}^{\prime }R)c_{\mathrm{crack}}^{\prime }R$ for $c_{\mathrm{crack}}^{\prime }R$ gives

2.6 $$c_{\mathrm{crack}}^{\prime }R=\frac{-cR+\sqrt{{\left(cR\right)}^{2}+4K^{2}}}{2}.$$

The solution with ‘−’ is discarded, since it results in a $c_{\mathrm{crack}}^{\prime }R<0$.

From equation (2.6) and *cR* ≥ 0, we obtain $cR+c_{\mathrm{crack}}^{\prime }R=\frac{cR+\sqrt{{\left(cR\right)}^{2}+4K^{2}}}{2}\geq K$. By definition $(cR+c_{\mathrm{crack}}^{\prime }R)c_{\mathrm{crack}}^{\prime }R=K^{2}$, it follows that $0<c_{\mathrm{crack}}^{\prime }R\leq K.$

#### 2.1.3. Derivation of $c_{\mathrm{bend}}^{\prime }R$ for bending failure

From equations (2.1) and (2.3), it follows that

2.7 $$\frac{M_{c^{\prime }}}{M_{\mathrm{bend}}}=\frac{c^{\prime }E_{L}R}{\sigma_{b}}.$$

Setting $M_{\mathrm{bend}}=M_{c^{\prime }}$ gives

2.8 $$c_{\mathrm{bend}}^{\prime }R=\frac{\sigma_{b}}{E_{L}}=\varepsilon_{L_{\text{-}\mathrm{failure}}},$$

where *ε_L-_*_failure_ is the normal strain at which the conventional bending failure occurs.

Note that equation (2.8) is independent of the bending direction, valid for both curvature-decreasing and curvature-increasing bending.

The failure mode can be determined by comparing equations (2.6) and (2.8)—$c_{\mathrm{bend}}^{\prime }R<c_{\mathrm{crack}}^{\prime }R$ for bending failure; otherwise, for cracking failure.

#### 2.1.4. Derivation of *Q* for determining whether the failure mode is bending or cracking

From equations (2.1) and (2.2), it follows that

2.9 $$\frac{M_{\mathrm{crack}}}{M_{\mathrm{bend}}}=\frac{2\sigma_{T}(t/R)}{3(cR+{c^{\prime }}_{\mathrm{crack}}R)\sigma_{b}{\left(1-(1/2)\;(t/R)\right)}^{3}},$$

where $c_{\mathrm{crack}}^{\prime }R$ is obtained from equation (2.6).

To determine the critical value, *cR* = *c_cri_R*, at which *M*_bend_ = *M*_crack_, we define $Q\equiv c_{cri}R+c_{\mathrm{crack}}^{\prime }R$ and from equation (2.9)

2.10 $$Q=\frac{2\sigma_{T}(t/R)}{3\sigma_{b}{\left(1-(1/2)\;(t/R)\right)}^{3}}.$$

Cracking failure occurs when *M*_crack_ < *M*_bend_, i.e. when *cR* > *c_cri_R* or $cR+c_{\mathrm{crack}}^{\prime }R>Q$; bending failure occurs when *M*_crack_ > *M*_bend_, i.e. when *cR* < *c_cri_R* or $cR+c_{\mathrm{crack}}^{\prime }R<Q$. As a result, a trunk with smaller *c_cri_* tends to fail due to cracking. At the critical condition of *M*_crack_ = *M*_bend_, $c_{}^{\prime }R=c_{\mathrm{crack}}^{\prime }R=c_{\mathrm{bend}}^{\prime }R$.

At *cR* = *c_cri_R*, $(c_{cri}R+c_{\mathrm{crack}}^{\prime }R)c_{\mathrm{crack}}^{\prime }R=Qc_{\mathrm{crack}}^{\prime }R=K^{2}$.

$$c_{\mathrm{crack}}^{\prime }R=\frac{K^{2}}{Q}=\frac{\sigma_{b}}{E_{L}}=c_{\mathrm{bend}}^{\prime }R$$

2.11 $$c_{cri}R=Q-c_{\mathrm{crack}}^{\prime }R=Q-\frac{\sigma_{b}}{E_{L}}\;\text{for }Q\geq K.$$

Note that equation (2.11) is only applicable for *Q* ≥ *K*. *Q* and *K* monotonically increase with *t*/*R*, and *Q* ≥ *K* when *t*/*R* ≥ (*t*/*R*)*_cri_*, where (*t*/*R*)*_cri_*, is the critical *t*/*R* ratio at which *Q* = *K* (electronic supplementary material, figure S1).

#### 2.1.5. Derivation of (*t*/*R*)*_cri_*

The critical ratio (*t*/*R*)*_cri_* is calculated by setting *Q* = *K*, using equations (2.5) and (2.10) as follows

2.12 $$\frac{{\left(t/R\right)}_{cri}}{{\left(1-(1/2){\left(t/R\right)}_{cri}\right)}^{3}}=\frac{3\sigma_{b}^{2}}{2E_{L}\sigma_{T}}.$$

### 2.2. Straight hollow trunk

For a straight trunk, the tangential cracking is initiated at *φ* = *π*/2 and the cracking moment *M*_crack0_ can be obtained from equation (2.2) by letting *cR* = 0 and $c_{crack}^{\prime }R=K$ as follows:

2.13 $$M_{\mathrm{crack}0}=\frac{I\sqrt{E_{L}\sigma_{T}(t/R)}}{R\sqrt{1.5{\left(1-(1/2)\;(t/R)\right)}^{3}}}.$$

The failure mode of a straight trunk can then be determined by comparing *M*_bend_ and *M*_crack_, which is the topic of our earlier work [13]. Note that equation (2.13) is slightly different from equation 2.13 of [13] and is more accurate since the point of action of *F* is changed from the periphery, i.e. *R*, to the centre of ring thickness *t*/2, i.e. *R* − *t*/2.

### 2.3. Initially curved hollow trunk subject to curvature-decreasing bending

#### 2.3.1. Derivation of *M*_crack_ for cracking failure

The bending moment *M*_crack_ at which the tangential cracking occurs in a curvature-decreasing bending can be derived by following the procedure similar to the curvature-increasing case, except that the cracking is initiated at *φ* = 0 (electronic supplementary material):

2.14 $$M_{\mathrm{crack}}=\frac{2\sigma_{T}I(t/R)}{3(cR-{c^{\prime }}_{\mathrm{crack}}R)R{\left(1-(1/2)\;(t/R)\right)}^{2}(1+(1/6)\;(t/R))}\;\text{for }\varphi =\text{0 and }c\gg c_{\mathrm{crack}}^{\prime },$$

where *cR* is the dimensionless initial curvature, and $c_{\mathrm{crack}}^{\prime }R$ the dimensionless change of curvature when cracking failure occurs, which will be determined in the following section.

#### 2.3.2. Derivation of *K* and $c_{\mathrm{crack}}^{\prime }R$ for cracking failure

From equations (2.3) and (2.14),

2.15 $$\frac{M_{c^{\prime }}}{M_{\mathrm{crack}}}=\frac{3(cR-{c^{\prime }}_{\mathrm{crack}}R){c^{\prime }}_{}RE_{L}{\left(1-(1/2)\;(t/R)\right)}^{2}(1+(1/6)\;(t/R))}{2\sigma_{T}(t/R)}\;\text{for }\varphi =0.$$

For crack failure initiation, $M_{c^{\prime }}=M_{\mathrm{crack}}$ and $c_{}^{\prime }R=c_{\mathrm{crack}}^{\prime }R$. Define $K\equiv \sqrt{(cR-{c^{\prime }}_{\mathrm{crack}}R){c^{\prime }}_{\mathrm{crack}}R}$ and from equation (2.15),

2.16 $$K=\frac{\sqrt{2\sigma_{T}(t/R)}}{(1-(1/2)\;tER)\sqrt{3E_{L}(1+(1/6)\;t/R)}}.$$

Solving $K^{2}=(cR-c_{\mathrm{crack}}^{\prime }R)c_{\mathrm{crack}}^{\prime }R$ for $c_{\mathrm{crack}}^{\prime }R$ gives

2.17 $$c_{\mathrm{crack}}^{\prime }R=\frac{cR\pm \sqrt{{\left(cR\right)}^{2}-4K^{2}}}{2}.$$

The solution with ‘+’ is discarded, since the smaller one will occur first. Then, we have $(cR-c_{\mathrm{crack}}^{\prime }R)\geq K\text{ and 0}\leq c_{\mathrm{crack}}^{\prime }R\leq K$.

Note that $c_{\mathrm{crack}}^{\prime }R$ exists if and only if (*cR*)^2^ ≥ 4*K*^2^ or *cR* ≥ 2*K*. The minimum value of *cR* is *c*_min_*R* = 2*K* and its corresponding $c_{\mathrm{crack}}^{\prime }R=K$.

#### 2.3.3. Derivation of *Q* for determining whether the failure mode is bending or cracking

From equations (2.1) and (2.14), it follows that

2.18 $$\frac{M_{\mathrm{crack}}}{M_{\mathrm{bend}}}=\frac{2\sigma_{T}(t/R)}{3(cR-{c^{\prime }}_{\mathrm{crack}}R)\sigma_{b}{\left(1-(1/2)\;(t/R)\right)}^{2}(1+(1/6)\;(t/R))},$$

where $c_{\mathrm{crack}}^{\prime }R$ is obtained from equation (2.17).

To determine the critical value, *cR* = *c_cri_R*, at which *M*_bend_ = *M*_crack_, we define $Q\equiv c_{cri}R-c_{\mathrm{crack}}^{\prime }R$ and from equation (2.18)

2.19 $$Q=\frac{2\sigma_{T}(t/R)}{3\sigma_{b}{\left(1-(1/2)\;(t/R)\right)}^{2}(1+(1/6)\;(t/R))}.$$

Cracking failure occurs when *M*_crack_ < *M*_bend_, i.e. when *cR* > *c_cri_R* or $cR-c_{\mathrm{crack}}^{\prime }R>Q$; bending failure occurs when *M*_crack_ > *M*_bend_, i.e. when *cR* < *c_cri_R* or $cR-c_{\mathrm{crack}}^{\prime }R<Q$. As a result, a trunk with smaller *c_cri_R* tends to fail due to cracking. At the critical condition of *M*_crack_ = *M*_bend_, $c_{}^{\prime }R=c_{\mathrm{crack}}^{\prime }R=c_{\mathrm{bend}}^{\prime }R$.

From equation (2.17) and *cR* ≥ *c*_min_*R* = 2 *K*, we obtain $cR-c_{\mathrm{crack}}^{\prime }R=cR+\sqrt{{\left(cR\right)}^{2}-4K^{2}}/2\geq K$

By definition $(cR-c_{\mathrm{crack}}^{\prime }R)c_{\mathrm{crack}}^{\prime }R=K^{2}$, it follows that $c_{\mathrm{crack}}^{\prime }R\leq K$.

At *c* = *c_cri_*, $(c_{cri}R-c_{\mathrm{crack}}^{\prime }R)c_{\mathrm{crack}}^{\prime }R=Qc_{\mathrm{crack}}^{\prime }R=K^{2}$.

$$c_{\mathrm{crack}}^{\prime }R=\frac{K^{2}}{Q}=\frac{\sigma_{b}}{E_{L}}=c_{\mathrm{bend}}^{\prime }R$$

and

2.20 $$c_{cri}R=Q+c_{\mathrm{crack}}^{\prime }R=Q+\frac{\sigma_{b}}{E_{L}}\;\text{for }Q\geq K.$$

Note that equation (2.20) is only applicable for *Q* ≥ *K*. *Q* and *K* monotonically increase with *t*/*R*, and *Q* ≥ *K* when *t*/*R* ≥ (*t*/*R*)*_cri_*, where (*t*/*R*)*_cri_*, is the critical *t*/*R* ratio at which *Q* = *K* (electronic supplementary material, figure S1).

#### 2.3.4. Derivation of (*t*/*R*)*_cri_*

The critical ratio (*t*/*R*)*_cri_* is calculated by setting *Q* = *K*, using equations (2.16) and (2.19) as follows

2.21 $$\frac{{\left(t/R\right)}_{cri}}{{\left(1-(1/2){\left(t/R\right)}_{cri}\right)}^{2}(1+(1/6){\left(t/R\right)}_{cri})}=\frac{3\sigma_{b}^{2}}{2E_{L}\sigma_{T}}.$$

## 3. Results and discussion

Equations (2.2) and (2.14) show that *M*_crack_ is proportional to the tangential tensile strength of wood *σ_T_*, increases with *t*/*R* and decreases with the final dimensionless curvature provided that *cR* ≥ 0 and *cR* ≥ 2 *K* for the curvature-increasing and curvature-decreasing conditions, respectively.

In this section, we describe case studies of several tree species using the equations presented in the previous section. Their mechanical properties, critical *t*/*R* ratios, *K* and *Q* for both curvature-increasing and curvature-decreasing cases are summarized in table 2.

**Table 2.** Mechanical properties, critical *t*/*R* ratios, *K* and *Q* for 11 green wood species [21].

species	Young's modulus (MPa)	bending strength (MPa)		critical *t*/*R* for straight trunks	*K* at *t*/*R* = 0.3		*Q* at *t*/*R* = 0.3	
	*E_L_*	*σ_b_*	*σ_T_*		curvature-increasing	curvature-decreasing	curvature-increasing	curvature-decreasing
*Zelkova serrata*^a^	9879	72	5.5	<0.11905	0.0135	0.0121	0.0249	0.0201
*Fagus crenata*^a^	9879	72	3.9	<0.15774	0.0113	0.0102	0.0176	0.0143
*Paulownia tomentosa*^a^	4212	22	1.5	<0.09872	0.0108	0.0097	0.0222	0.0180
*Quercus mongolica*^a^	8835	72	4.7	<0.14856	0.0132	0.0118	0.0213	0.0172
*Terminalia* sp.^c^	7903	49	2.9	<0.12871	0.0109	0.0098	0.0193	0.0156
*Shorea* spp.^c^	9320	52	2.3	<0.14980	0.0090	0.0081	0.0144	0.0117
*Dipterocarpus grandiflorus*^c^	14 166	85	2.5	<0.21687	0.0076	0.0068	0.0096	0.0078
*Cryptomeria japonica*^b^	5777	46	1.2	<0.28745	0.0082	0.0074	0.0085	0.0069
*Picea jezoensis*^b^	7269	46	1.7	<0.19029	0.0087	0.0079	0.0120	0.0097
*Pinus densiflora*^b^	9067	64	1.6	<0.27279	0.0076	0.0068	0.0081	0.0066
*Agathis* spp.^c^	9067	36	1.6	<0.11262	0.0076	0.0068	0.0145	0.0117

^a^Hardwood.

^b^Softwood.

^c^Tropical wood.

We first compare the bending moment at which the tangential crack occurs under curvature-increasing and curvature-decreasing cases by evaluating *M*_dec_/*M*_inc_, where the subscript denotes the bending direction. Using *Zelkova serrata* as an example, we find that, for larger *cR*, cracking failure is easier to occur for curvature decrease than curvature increase, i.e. *M*_dec_/*M*_inc_ < 1, due to additional normal tensile force *F* acting on the neutral cross-section; on the other hand, for smaller *cR* bending failure is easier to occur due to decreased final curvature (electronic supplementary material, figure S2).

**Figure 3.**
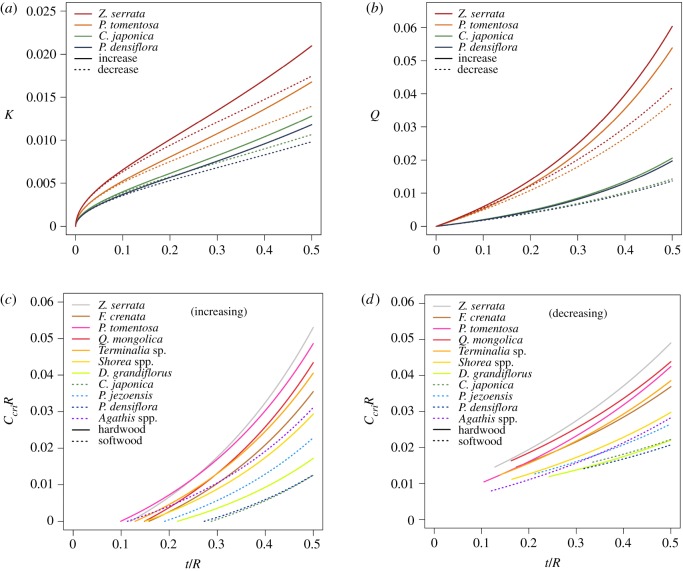
*K*, *Q* and *c_cri_R* as a function of *t*/*R* for various tree species subject to curvature-increasing and curvature-decreasing bending. Equations used to compute the results are equations (2.5) and (2.16) for (*a*), equations (2.10) and (2.19, for (*b*), equation (2.11) for (*c*) and equation (2.20) for (*d*).

Figure 3 compares *K*, *Q* and *c_cri_R* for various tree species. We observe that all parameters increase with *t*/*R*. Figure 3*a* shows *K* of two hardwood and two softwood species under curvature-increasing or curvature-decreasing bending. An investigation of the result highlights two key observations. First, curvature-increasing bending has larger *K* than the case of curvature-decreasing. This is due to the additional tensile stress *F*/*t* exerted on the cracking plane, *φ* = 0, of the curvature-decreasing case (figure 2*f*), where *F* is the internal normal force per unit length derived in equation S2 of electronic supplementary material. Such a tensile stress does not exist on the cracking plane, *φ* = *π*/2, of the curvature-increasing case (figure 2*e*). Second, the hardwoods in general have larger *K* than the softwoods. Recall that *M*_crack_ is proportional to $c_{\mathrm{crack}}^{\prime }R$ since $M_{\mathrm{crack}}=M_{c^{\prime }}=c_{\mathrm{crack}}^{\prime }E_{L}I$.

Figure 3*b* shows *Q* of the four species under curvature-increasing or curvature-decreasing bending. We observe a trend similar to *K* that is the hardwoods, such as *Z*. *serrata* and *Paulownia tomentosa*, have larger *Q* than that of the softwoods, such as *Cryptomeria japonica* and *Pinus densiflora*. Curvature-increasing bending has larger *Q* than the case of curvature-decreasing. At such a condition, hardwood is in general stronger in resisting cracking failure than softwood.

When given the geometrical and material properties of a tree, we may use *c_cri_R* as derived in equations (2.11) and (2.20) to determine the mode of failure—*cR* > *c_cri_R* for cracking failure and *cR* < *c_cri_R* for bending failure. Figure 3*c*,*d* shows *c_cri_R* for 11 tree species, including four hardwoods, three softwoods and four tropical woods, under curvature-increasing or curvature-decreasing bending, respectively. Note that tropical woods are in general hardwoods, but one of the species used here, *Agathis* spp., is a softwood. Recall that *c_cri_* is the critical initial curvature at *M*_bend_ = *M*_crack_, which exists if and only if *Q* ≥ *K* or *t*/*R* ≥ (*t*/*R*)*_cri_*. At *t*/*R* = (*t*/*R*)*_cri_*, *c_cri_R* = 0 and = 2*σ_b_*/*E_L_*, respectively, for the case of curvature-increasing and curvature-decreasing. The hardwoods tend to have larger *c_cri_R* than the softwoods and tropical woods, indicating that at a given *t*/*R* and *cR*, hardwoods are more resistant to cracking failure (*cR* < *c_cri_R* or *M*_bend_ < *M*_crack_), compared with softwoods and tropical woods, which are more likely to experience cracking failure (*cR* > *c_cri_R* or *M*_bend_ > *M*_crack_).

No data point exists for *t*/*R* < (*t*/*R*)*_cri_* in curvature-decreasing case (figure 3*d*), which means that bending and cracking failures cannot both exist. However, failure may still occur within this range, depending on the conditions as follows: (i) cracking only, if *cR* ≥ 2 *K*, (ii) no cracking, if *cR* < 2 *K*, (iii) bending failure only, if *cR* < 2 *K* and *σ_b_*/*E_L_* < *cR* ≤ 2*σ_b_*/*E_L_*, and (iv) no failure, if *cR* < 2 *K* and 0 < *cR* < *σ_b_*/*E_L_*. Here, we assume that the trunk does not flip over, i.e. $c_{}^{\prime }R\leq cR$.

**Figure 4.**
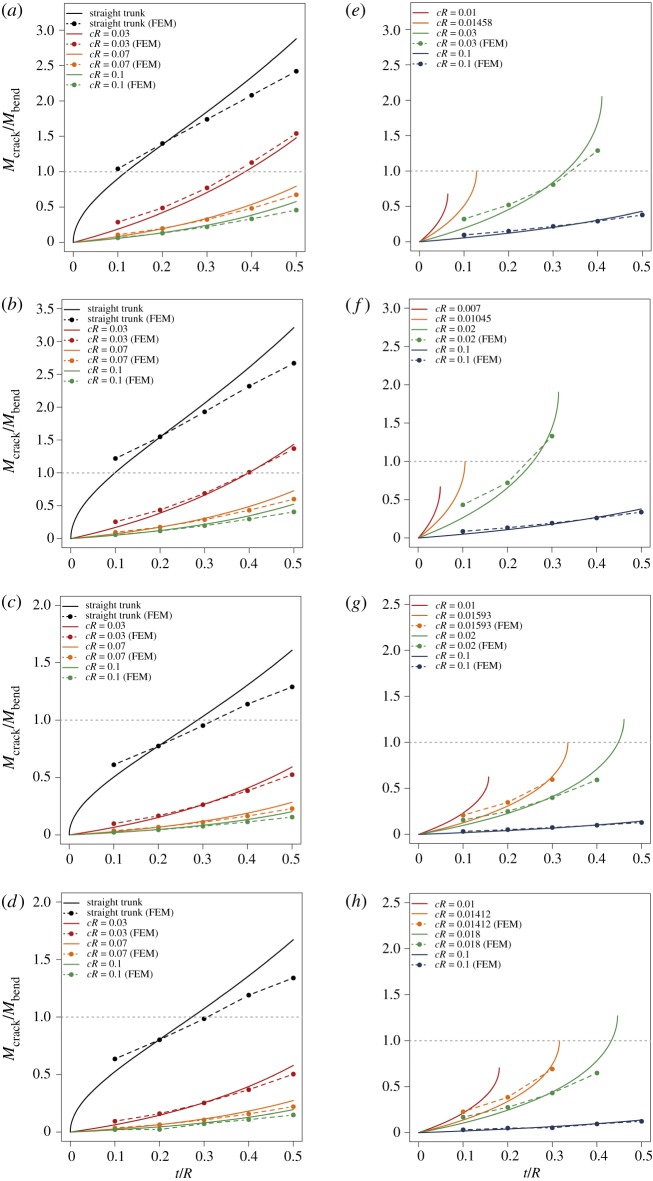
*M*_crack_/*M*_bend_ as a function of *t*/*R* for various tree species subject to curvature-increasing bending (*a*−*d*) (equation 2.9) and curvature-decreasing bending (*e*−*h*) (equation 2.18). (*a*,*e*) *Z. serrata*. (*b*,*f*) *P. tomentosa*. (*c*,*g*) *C. japonica*. (*d*,*h*) *P. densiflora*. *M*_crack_/*M*_bend_ > 1 indicates that the trunk fails at bending and *M*_crack_/*M*_bend_ < 1 fails at cracking. The finite-element method (FEM) results were obtained by the commercial simulation package, Abaqus.

Figure 4 shows *M*_crack_/*M*_bend_ as a function of *t*/*R* for four species. Bending failure occurs when *M*_crack_/*M*_bend_ > 1, otherwise cracking failure occurs. For instance, for *Z*. *serrata* with *cR* = 0.03, the critical (*t*/*R*)*_cri_* is 0.39, indicating that the trunk will fail due to cracking as *t*/*R* < 0.39 and due to conventional bending as *t*/*R* > 0.39 (figure 4*a*). Also, at a given *t*/*R*, the larger *cR*, the more likely the cracking failure will occur. Unlike the curvature-increasing case, where failure will always occur as $M_{c^{\prime }}$ increases, a trunk subjected to curvature-decreasing bending may not fail all the way to $cR-c_{}^{\prime }R=0$, i.e. a straightened trunk. As shown in figure 4*e*, for *Z*. *serrata* with *cR* = 0.01, the trunk fails due to cracking as *t*/*R* < 0.065 and due to bending as *t*/*R* > 0.065. Although no data are shown for *t*/*R* > 0.065 since *M*_crack_ does not exist, the trunk still fails in bending as long as *cR* ≥ *σ_b_*/*E_L_* = 0.0073; similarly, for *cR* = 0.03, the trunk fails due to cracking as *t*/*R* < 0.33 and due to bending as *t*/*R* > 0.33. For the special case *cR* = *c_cri_R* = 0.0145 and (*t*/*R*)*_cri_* = 0.128, where *Q* = *K*, the trunk fails due to cracking as *t*/*R* less than 0.128 and fails in bending as *t*/*R* > 0.128. Similar observations can be made for the other three species. We find that, compared with the two hardwood species, the two softwood species are more likely to fail in cracking for a given *t*/*R* and *cR*, which is consistent with the observations in figure 3.

**Figure 5.**
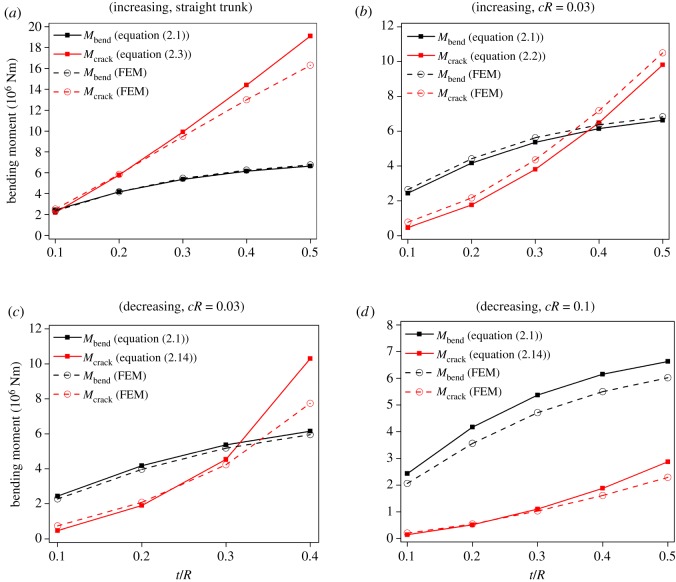
Comparison of *M*_bend_ and *M*_crack_ of *Zelkova serrata* between the analytical and the FEM results. (*a*,*b*) Curvature-increasing bending and (*c*,*d*) curvature-decreasing bending.

We also compare the theoretical predictions with those from the finite-element method (FEM) simulation (figures 4 and 5). This serves as a check on any errors in the mechanics theory, though it may not be a substitute for a comparison with experimental data. The results from both methods are in general consistent. The deviation is relatively large for large *t*/*R*, e.g. ≥0.5, and for small *t*/*R*, e.g. ≤0.1. The former is due to the fact that d*F* is assumed to act at *R* − *t*/2 (figure 2*e*,*f*), which introduces certain error for large *t*/*R*, in which case the radial coordinate of the centroid of the shaded trapezoid is slightly greater than *R* − *t*/2. The latter is due to the fact that our FEM simulation considers the Brazier buckling effect (figure 1*c*,*d*), which is not considered in our theoretical models. Brazier buckling is known to become the dominant failure mode for very small *t*/*R* [13].

**Figure 6.**
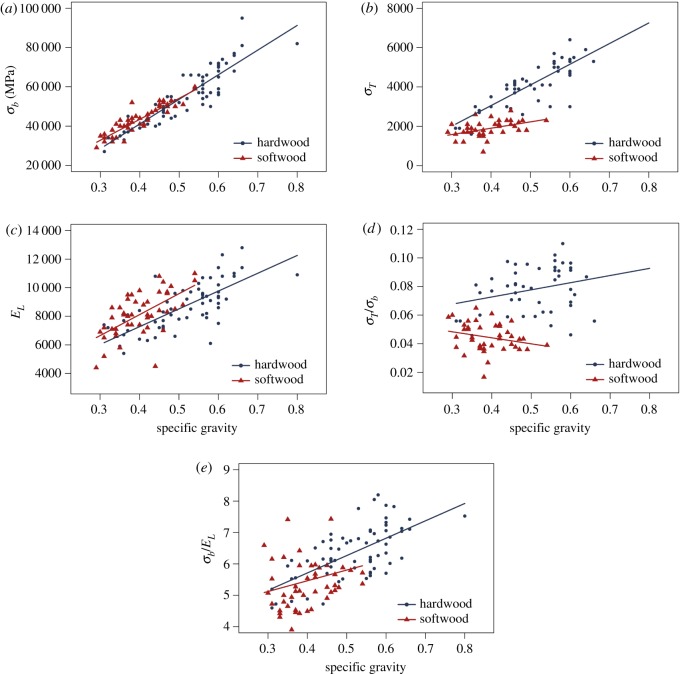
Comparison of material properties, as a function of specific gravity, between hardwoods and softwoods for 66 species of hardwoods and 43 species of softwoods. (*a*) Bending strength, (*b*) tangential component of tensile strength perpendicular to grain, or tangential tensile strength for short, (*c*) Young's modulus in the longitudinal direction, (*d*) ratio of the tangential strength to the bending strength and (*e*) ratio of the bending strength to the Young's modulus.

A particular hollow trunk's failure mode is determined by its geometric parameters and material properties, including Young's modulus, bending strength and the tangential tensile strength. To confirm whether the trends observed in figures 3 and 4 are still valid for a larger number of species, we expand our analysis to include 66 hardwoods and 43 softwoods (figure 6) with the green wood properties from *Wood Handbook—Wood as an Engineering Material* [19]. Bending strength (*σ_b_*), tangential tensile strength (*σ_T_*), Young's modulus (*E_L_*) and their ratios are plotted against specific gravity, since specific gravity is highly correlated with wood's strength and elasticity [20]. For the species analysed here, the specific gravity of hardwoods ranges approximately from 0.31 to 0.80, whereas that of softwoods ranges approximately from 0.29 to 0.54. *E_L_*, *σ_b_* and *σ_T_* increase with specific gravity for both hardwoods and softwoods. Softwoods, however, have significantly lower tangential strength than the hardwoods with similar specific gravity. *σ_T_*/*σ_b_* of the softwoods is significantly smaller than that of hardwoods, and they exhibit distinct patterns—the ratio increases with specific gravity for hardwoods and decreases for softwoods. Since *Q* is proportional to *σ_T_*/*σ_b_* and *c_cri_R* increases with *Q*, softwoods have smaller *σ_T_*/*σ_b_* and *c_cri_R*, indicating that softwoods are more likely to fail in cracking. This reaffirms the findings shown in figures 3 and 4. Also, we note that the failure mode of hardwoods and softwoods show an opposite trend as a function of specific gravity. The difference in the mechanical properties, and hence the failure mode, between softwoods and hardwoods, might be explained by their different microstructures [22]. Softwoods, such as Japanese cedar (*C. japonica*), consist of up to 95% tracheids oriented along the trunk. Only approximately 5% of the tissue is oriented in the radial direction, the uniseriate rays. By contrast, hardwoods, such as Japanese zelkova (*Z. serrata*), have more complex microstructures, consisting of different tissue types, such as vessels, fibre tracheids, libriform fibres and rays. Hardwoods can have larger rays (multiseriate rays) and a higher relative volume fraction of such radially oriented tissue—typically 12% for alder, 20% for oak and 16% for ash. There are no fibres and rays aligning along the tangential direction that serve as strengthening mechanisms [23].

During secondary growth, xylem accumulates growth stresses that are similar to the residual stresses that occur in artificial materials during processing, such as thermal stress. These growth stresses represent an important bio-mechanical mechanism that enables the tree to adjust to the ecological environment [24–26]. In coniferous trees, compression wood is formed on the lower side of a tilted trunk (longitudinal compressive stress), whereas in dicotyledonous trees, tension wood is formed on the upper side (longitudinal tensile stress). The tangential growth stresses are always compressive. By producing reaction wood, up-righting of leaning trunks or branches is achieved. These growth stresses can influence the precision of *M*_crack_ calculation. However, very few data are available. On the other hand, in the hollowing process, a large part of the growth stresses can be released, and hence the effect of growth stresses on cracking will be reduced. Therefore, in this study, the effect of growth stress is neglected. Also, we use unidirectional strength criteria—we neglect the fact that tangential stress on wood may modify its longitudinal strength.

How important is it to account for initial curvature, compared with the straight case, i.e. equation (2.13)? What is the threshold *cR* above which initial curvature can no longer be neglected? Suppose an error of 10% is acceptable for the curvature-increasing case, from equations (2.2) and (2.13), we have $M_{\mathrm{crack}0}/M_{\mathrm{crack}}=(cR+c_{\mathrm{crack}}^{\prime }R)/K=1.1$ or $cR+c_{\mathrm{crack}}^{\prime }R=1.1K$. Recall that $(cR+c_{\mathrm{crack}}^{\prime }R)c_{\mathrm{crack}}^{\prime }R\equiv K^{2}$. It follows that $c_{\mathrm{crack}}^{\prime }R=K^{2}/1.1K\approx 0.91K$. The threshold *cR* for 10% error is then determined to be *cR* = 1.1*K* − 0.91*K* = 0.19*K*, where *K* can be obtained from equation (2.5) or figure 3*a*.

## 4. Conclusion

In this paper, we investigate the failure modes of curved hollow trunks due to bending by comparing the tangential cracking (longitudinal splitting) and conventional bending failure. We derive new analytical expressions for predicting the bending moment at which tangential cracking occurs under the curvature-decreasing and curvature-increasing bending. We also derive analytical expressions for critical thickness to radius ratio (*t*/*R*)*_cri_* and critical *c_cri_R*, which can be used to predict the failure mode of a trunk with given geometric parameters and material properties. We study 11 tree species and find that the hardwoods are more likely to break due to bending failure, whereas softwoods and tropical woods tend to break due to cracking failure. This can be attributed to the softwoods' much smaller tangential tensile strength, compared with the hardwoods, based on the data of 66 hardwoods and 43 softwoods. At the same specific gravity, softwoods have a similar bending strength as hardwoods, but their tangential tensile strength is significantly lower. A trunk subjected to a curvature-increasing moment will eventually crack or fail in bending. By contrast, for the curvature-decreasing case, whether or not the trunk fails and in which mode depend on its *cR*, *t*/*R* and material properties. For larger *cR*, cracking failure is easier to occur for curvature decrease than curvature increase due to additional normal tensile force *F* acting on the neutral cross-section; on the other hand, for smaller *cR* bending failure is easier to occur due to decreased final curvature. Our findings may be readily applied to assess the failure potential of other natural and man-made curved hollow structures with orthotropic material properties and may shed light on the safety assessment, conservation and ecology of trees.

## Supplementary Material

Detailed theoretical formulation of cracking moment

Reviewer comments

## Data accessibility

The datasets for generating the table and figures have been uploaded as electronic supplementary material, alongside this article.
