# Correction to ‘Cracking failure of curved hollow tree trunks’

**DOI:** 10.1098/rsos.200643

**Published:** 2020-05-13

**Authors:** Yan-San Huang, Pei-Lin Chiang, Ying-Chuan Kao, Fu-Lan Hsu, Jia-Yang Juang

*R. Soc. Open Sci.* **7**, 200203 (Published Online 11 March 2020) (doi:10.1098/rsos.200203)

This correction refers to an error in equation 2.16, *tER* was used in place of *t*/*R* (see below). This has now been updated.

**Corrected version**

$$K=\frac{\sqrt{2\sigma_{T}(t/R)}}{(1-(1/2)\;(t/R))\sqrt{3E_{L}(1+(1/6)\;(t/R))}}$$

