# Supplementary material for: Cracking failure of curved hollow tree trunks
Source: R Soc Open Sci. 2020 Mar 11;7(3):200203. doi: 10.1098/rsos.200203 (PMC7137967; doi:10.1098/rsos.200203)
Supplement: Detailed theoretical formulation of cracking moment [file rsos200203supp1.pdf]

# Supplementary Material

## Cracking failure of curved hollow tree trunks

**Yan-San Huang[1*], Pei-Lin Chiang[2], Ying-Chuan Kao[2], Fu-Lan Hsu[3], and Jia-Yang Juang[2*]**

[1]Department of Forestry
National Chung Hsing University
Taichung 402, Taiwan (R.O.C.)
[2]Department of Mechanical Engineering
National Taiwan University
Taipei 10617, Taiwan (R.O.C.)
[3]Division of Forest Chemistry
Taiwan Forestry Research Institute
Taipei 10066, Taiwan (R.O.C.)

To whom correspondence may be addressed.

Email: Y.-S.H: yansanhuang@ntu.edu.tw or J.-Y.J: jiayang@ntu.edu.tw

## Theoretical Formulation

### (a) Derivation of $M_{\text{crack}}$ for curved hollow trunk subject to curvature-decreasing bending

We follow the procedure presented in our earlier work to derive the bending moment for cracking failure [1]. Figure 2*b* shows the schematic of initially curved hollow trunk subject to curvature-decreasing bending. The bending deformation of the trunk induces longitudinal compressive stress on the convex side and tensile stress on the concave side of the trunk, assuming that the change of curvature, $c'$ , is smaller than the initial curvature, *c*, so that the trunk does not flip over. The tensile and compressive stresses both create transverse outward forces pulling away from the *x* axis, i.e., the neutral axis (figure 2*d*). These transverse forces are body forces and tend to flatten the cross section to form an oval with its long axis lying on the *y* axis (figure 2*b* and figure 1*d*). The outward force d*F* per unit axial length and a circumferential width of $R\mathrm{d}\theta$ set up by the longitudinal bending stress, $\sigma_L$ , induced by a bent trunk with a final curvature $c_f = c - c'$

$$dF = c_f \frac{M_{c'}}{I} R^2 \left(1 - \frac{1}{2}\frac{t}{R}\right)^2 t \sin\theta d\theta \tag{S1}$$

where $M_{c'}$ is the bending moment that creates a curvature change of $c'$.

Due to symmetry only one quarter of the ring is considered. The internal normal force per unit length *F* is

$$F = \int_0^{\frac{\pi}{2}} dF = c_f \frac{M_{c'}}{I} R^2 (1 - \frac{1}{2}\frac{t}{R})^2 t \tag{S2}$$

The magnitude of the tangential bending moment $M_0$ per unit length acting on the axial-radial plane at $\varphi = 0$ is statically indeterminate, and can be calculated using the Castigliano's theorem. By using $M(\varphi)$ as the moment of unit length at an angle $\varphi$ from the *x* axis (figure 2*f*) and $I_z$ as the moment of inertia of unit length of the cross section, the strain energy *U* per unit length for one quarter of the section is

$$U = \int_0^{\frac{\pi}{2}} \frac{M(\varphi)^2 R(1 - \frac{1}{2}\frac{t}{R}) d\varphi}{2E_{\mathrm{T}} I_z} \tag{S3}$$

and the moment $M(\varphi)$ is given by

$$\begin{aligned} M(\varphi) &= M_0 - FR(1 - \frac{1}{2}\frac{t}{R})(1 - \cos\varphi) + \int_0^{\varphi} FR(1 - \frac{1}{2}\frac{t}{R})(\cos\theta - \cos\varphi)\sin\theta d\theta \\ &= M_0 - \frac{1}{2} FR(1 - \frac{1}{2}\frac{t}{R})(1 - \cos^2\varphi) \end{aligned} \tag{S4}$$

Owing to the condition of symmetry the cross section at $\varphi = 0$ does not rotate during bending, such that

$$\frac{\partial U}{\partial M_0} = 0 \tag{S5}$$

Because $\partial M(\varphi)/\partial M_0 = 1$, we have

$$\frac{\partial U}{\partial M_0} = \frac{1}{E_T I_z}\int_0^{\frac{\pi}{2}}\left[M_0 - \frac{1}{2}FR(1-\frac{1}{2}\frac{t}{R})(1-\cos^2\phi)\right]R(1-\frac{1}{2}\frac{t}{R})d\phi = 0 \tag{S6}$$

then

$$M_0 = \frac{1}{4}FR(1-\frac{1}{2}\frac{t}{R}) \tag{S7}$$

Substituting for $M_0$ from equation (S7) into equation (S4), we obtain

$$M(\varphi) = \frac{1}{2}FR(1-\frac{1}{2}\frac{t}{R})(\cos^2\varphi - \frac{1}{2}) \tag{S8}$$

And $M(0) = M_0 = \frac{1}{4}FR(1-\frac{1}{2}\frac{t}{R})$; $M(\frac{\pi}{2}) = -\frac{1}{4}FR(1-\frac{1}{2}\frac{t}{R})$

At $\varphi = 0$, the normal stress distribution on the axial-radial plane can obtained by superposing the stress ($\sigma_1$) due to the normal force $F$ and the bending stress ($\sigma_2$) due to the bending moment $M_0$ as follows

$$\sigma = \sigma_1 + \sigma_2 = \frac{6M_0}{t^2} + \frac{F}{t} = \frac{6FR\left(1-\frac{1}{2}\frac{t}{R}\right)}{4t^2} + \frac{F}{t} \tag{S9}$$

Tangential cracking occurs when $\sigma = \sigma_T$, the tangential component of tensile strength perpendicular to grain, or tangential tensile strength for short.

Equation (S9) is rearranged as

$$F\left(\frac{3R(1-\frac{1}{2}\frac{t}{R})}{2t^2} + \frac{1}{t}\right) = \sigma_T \tag{S10}$$

Substituting for $F$ from equation (S2) into equation (S10), we obtain the bending moment $M_{c'} = M_{\text{crack}}$ at which the tangential cracking is initiated at $\varphi = 0$

$$M_{\text{crack}} = \frac{\sigma_T I(\frac{t}{R})}{1.5c_f R^2(1-\frac{1}{2}\frac{t}{R})^2(1+\frac{1}{6}\frac{t}{R})} = \frac{2\sigma_T I(\frac{t}{R})}{3(cR - c'_{\text{crack}}R)R(1-\frac{1}{2}\frac{t}{R})^2(1+\frac{1}{6}\frac{t}{R})} \quad \text{for } \varphi = 0 \text{ and } c >> c'_{\text{crack}} \tag{S11}$$

Note that equation (S11) is equation (2.14) in the main text.

**(b) Derivation of $M_{\mathrm{crack}}$ for curved hollow trunk subject to curvature-increasing bending**

Figure 2*a* shows the schematic of initially curved hollow trunk subject to curvature-increasing bending. The bending deformation of the trunk induces longitudinal tensile stress on the convex side and compressive stress on the concave side of the trunk. The tensile and compressive stresses both create transverse inward forces toward the *x* axis, i.e., the neutral axis (figure 2*c*). These transverse forces are body forces and tend to flatten the cross section to form an oval with its long axis lying on the *x* axis (figure 2*a* and figure 1*c*). The inward force d*F* per unit axial length and a circumferential width of $R\mathrm{d}\theta$ set up by the longitudinal bending stress, $\sigma_L$, induced by a bent trunk with a final curvature $c_f = c + c'$ can also be expressed by equation (S1). Following the procedure presented above for the curvature-decreasing case, we obtain the bending moment $M_{c'}$=$M_{\mathrm{crack}}$ at which the tangential cracking is initiated at $\varphi = \pi/2$

$$M_{\mathrm{crack}} = \frac{\sigma_T I(\frac{t}{R})}{1.5c_f R^2(1-\frac{1}{2}\frac{t}{R})^3} = \frac{2\sigma_T I(\frac{t}{R})}{3(cR + c'_{\mathrm{crack}}R)R(1-\frac{1}{2}\frac{t}{R})^3} \quad \text{for } \varphi = \frac{\pi}{2} \qquad \text{(S12)}$$

Note that equation (S12) is equation (2.2) in the main text. Unlike the curvature-decreasing case, the tangential cracking in this case is initiated at $\varphi = \pi/2$ and does not have a contribution from the normal force *F*.

**References**

1. Huang Y.S., Hsu F.L., Lee C.M., Juang J.Y. 2017 Failure mechanism of hollow tree trunks due to cross-sectional flattening. *R Soc Open Sci* **4**(4), 160972. (doi:10.1098/rsos.160972).

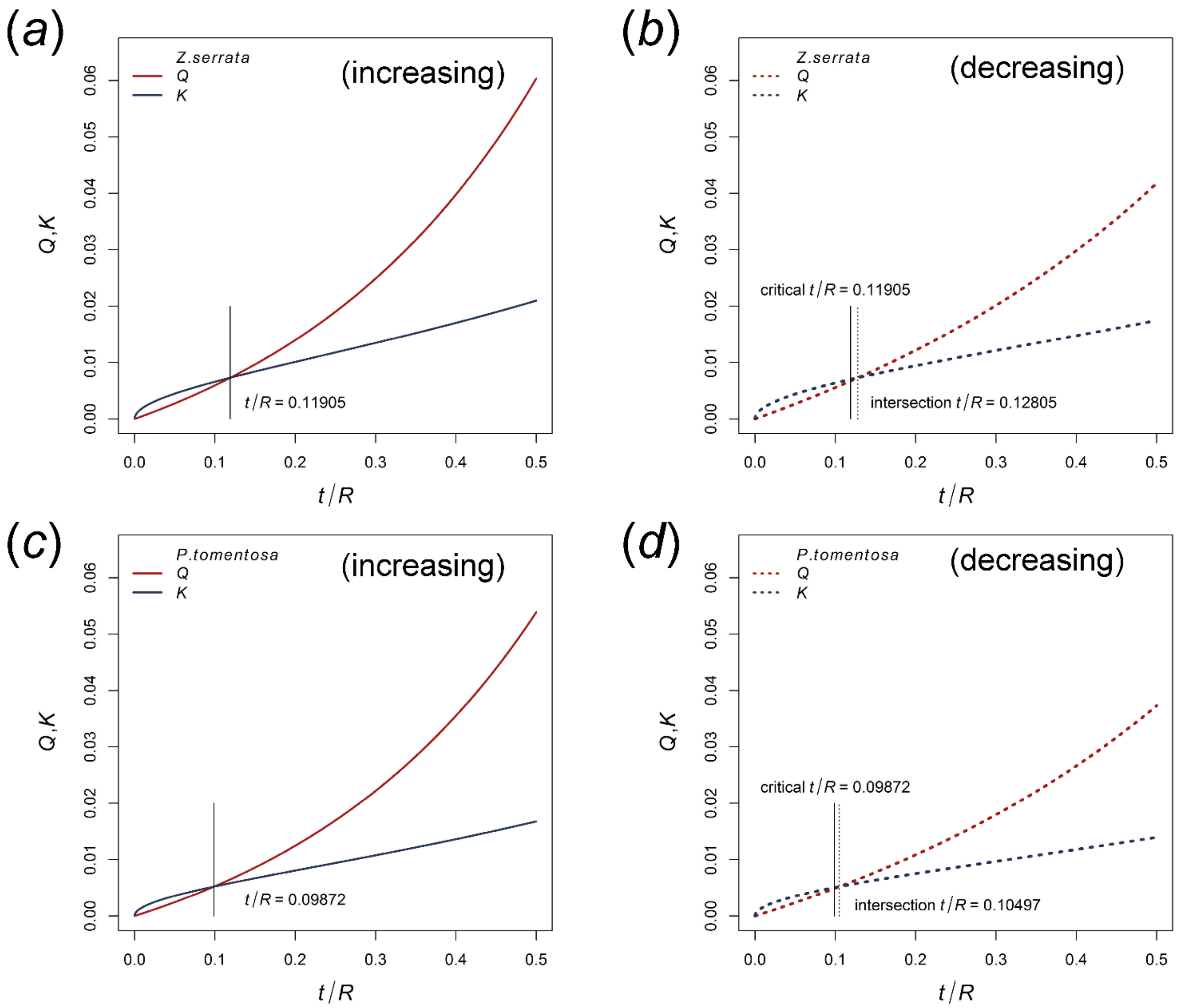


**Figure S1.** $K$ and $Q$ as a function of $t/R$ subject to curvature-increasing and curvature-decreasing bending. $Q > K$ when $t/R > (t/R)_{cri}$. Tree species: (*a*), (*b*) *Z. serrata*. (*c*), (*d*) *P. tomentosa*.

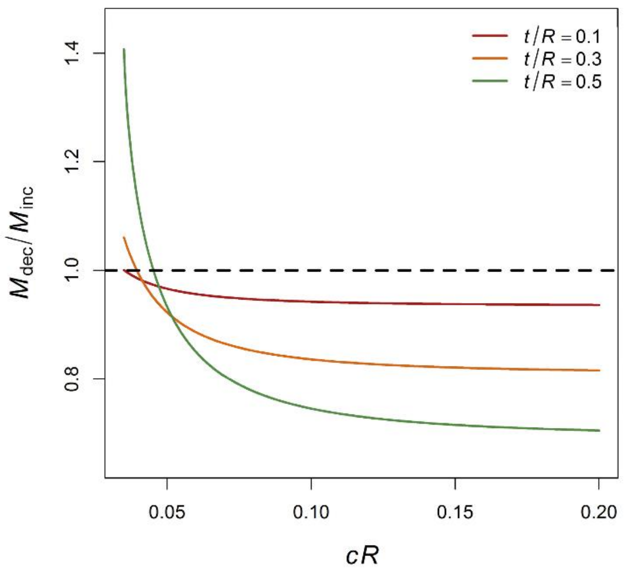


**Figure S2.** Comparison of the cracking moment under the curvature-increasing and curvature-decreasing loading. Tree species: *Zelkova serrata*.
